# Knee Osteoarthritis and Metabolic Disorders: Practical Aspects from Primary Healthcare

**DOI:** 10.3390/jcm15176906

**Published:** 2026-09-07

**Authors:** Natalia Kasprzyk, Paweł Jagielski, Marta Stelmach-Mardas, Ewa Lucka, Ewelina Kowynia, Bogna Grygiel-Górniak

**Affiliations:** 1 Department of Rheumatology, Rehabilitation and Internal Diseases, Poznan University of Medical Sciences, 60-545 Poznan, Poland; 2 Department of Nutrition and Drug Research, Institute of Public Health, Faculty of Health Sciences, Jagiellonian University Medical College, 31-066 Kraków, Poland; 3 Department of Medical Education and Communication, Poznan University of Medical Sciences, 60-806 Poznan, Poland; 4 Clinical Rehabilitation Laboratory, Department of Rehabilitation and Physiotherapy, Poznan University of Medical Sciences, 60-545 Poznan, Poland

**Keywords:** knee osteoarthritis, metabolic parameters, anthropometric measurement, nutrition

## Abstract

**Background/Objectives**: Knee osteoarthritis (KOA) is one of the most common musculoskeletal disorders worldwide, causing pain and disability. In addition to mechanical overload, increasing evidence suggests that metabolic disorders and abdominal obesity may contribute to the pathogenesis and progression of OA through mild inflammation and metabolic dysregulation. **Methods**: This cross-sectional, single-center study included 108 patients with radiologically confirmed KOA and 33 control subjects aged 45–80 years. KOA severity was assessed using the Kellgren–Lawrence scale and the Osteoarthritis Research Society International (OARSI) scale. Anthropometric and metabolic measurements, namely body mass index (BMI), waist-to-height ratio (WHtR), body shape index (ABSI), body roundness index (BRI), lipid accumulation product (LAP), visceral adiposity index (VAI), and dysfunctional obesity index (DAI), were analyzed. Correlation and multivariate regression analyses were performed to identify factors associated with KOA severity. **Results**: Over 85% of KOA patients were overweight or obese, and 63.9% met the criteria for metabolic syndrome. KOA patients had a significantly higher BMI, WHtR, waist circumference, BRI, LAP, and a higher prevalence of obesity than the control group. WHtR, BRI, and serum glucose levels positively correlated with OARSI scores. In multivariate linear regression analysis, age remained the only independent predictor of higher OARSI scores. Saturated fat intake exceeded recommended levels in both groups, but macronutrient intake was not associated with KOA severity. **Conclusions**: Age is the strongest independent predictor of structural KOA severity; however, visceral fat distribution and serum glucose levels correlate with the OARSI score, and modifying them may help improve cardiometabolic health in patients with KOA.

## 1. Introduction

Osteoarthritis (OA) is the most common form of arthritis and one of the leading causes of morbidity and functional disability worldwide. It is characterized by progressive cartilage degradation, joint stiffness, and pain [1,2]. The knee joint is a common site of degenerative changes. According to the Global Burden of Disease, Injuries, and Risk Factors Study (GBD), approximately 374.7 million people worldwide suffered from OA in 2021, with 30.8 million new cases reported annually. Furthermore, the incidence of this disease has been increasing since 1990, primarily due to the aging population [3].

OA is primarily considered an age-related disease. However, excess body weight accelerates its progression by increasing the mechanical stress on joints, leading to cartilage damage [4,5]. In recent years, a growing body of evidence has emerged suggesting a strong association between metabolic syndrome (MetS) and OA [6,7,8]. Understanding the role of metabolic factors in joint cartilage damage has led to the concept of a metabolic phenotype in OA, in which individual components of the MetS may independently contribute to the progression of cartilage degeneration. Furthermore, many anthropometric and lipid parameters, such as the body shape index (ABSI), visceral adiposity index (VAI), lipid accumulation product (LAP), dysfunctional adiposity index (DAI), and body roundness index (BRI), have been used to describe metabolic risk, highlighting the relationship between visceral adiposity distribution and metabolic risk [9,10,11].

Several mechanisms have been proposed to explain the association between MetS and OA. First, MetS is associated with chronic, mild inflammation, which may accelerate cartilage degeneration by increasing the production of proinflammatory cytokines and adipokines. Secondly, oxidative stress and metabolic disorders, including insulin resistance, may contribute to chondrocyte dysfunction and structural changes in joint cartilage [12,13]. The above risk factors suggest a shift in thinking from emphasizing only increased body weight and, consequently, mechanical stress in the development of KOA to considering whether visceral fat may be related to the severity of degenerative changes in the knee joints.

Taking the above into account, we decided to assess the relationships among metabolic factors, anthropometric, biochemical, and nutritional parameters in a group of KOA patients attending a primary health care clinic.

## 2. Materials and Methods

### 2.1. Study Group

This single-center cross-sectional study included 108 participants (78 women and 30 men) aged 45–80 years. All participants were diagnosed with KOA based on clinical symptoms and radiographic findings, as assessed by two independent rheumatologists. KOA was graded according to the Kellgren–Lawrence grading system (grades 0–4), with grade 1 representing questionable joint space narrowing and possible osteophytic bulging; grade 2 characterized by definite osteophytes with possible joint space narrowing; grade 3 representing multiple osteophytes, definite joint space narrowing, bone sclerosis, and possible bone deformity; and grade 4 with typical large osteophytes, marked joint space narrowing, severe subchondral sclerosis, and definite bone deformity [14]. In addition, the Osteoarthritis Research Society International (OARSI) atlas was used to assess osteophyte formation and joint space narrowing in the medial and lateral tibiofemoral compartments. All components were assessed to determine the final OARSI score [15].

**Figure 1:**
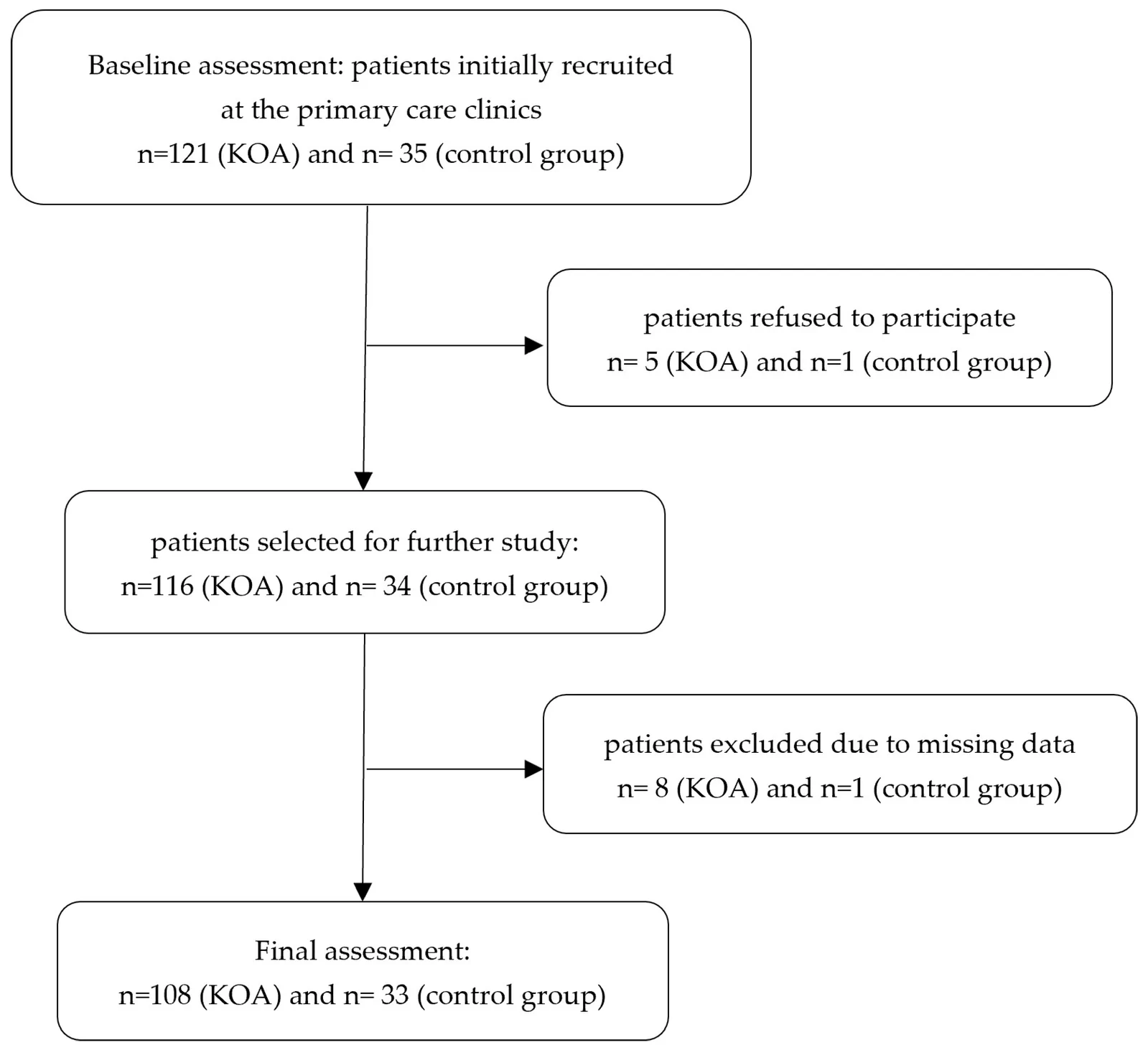
Flowchart of patients’ selection (KOA—knee osteoarthritis).

Patients were recruited from primary care clinics (n = 121 in the KOA group and n = 35 in the control group). After final selection, 108 participants with KOA and 33 healthy controls were included in the study (Figure 1). Data on general characteristics, including age, gender, smoking status, supplement use, disease duration, and medical history, were collected using a self-administered questionnaire. Participants with rheumatic diseases other than KOA, acute infections at the time of the study, a history of malignancy, or conditions that may affect basal metabolic rate (e.g., untreated hyperthyroidism or hypothyroidism, Cushing’s syndrome, renal and hepatic dysfunction) were excluded from the study. The study protocol was approved by the Bioethical Committee at the Poznan University of Medical Sciences (KB-854/23). All enrolled participants provided written informed consent. The study was conducted in accordance with the Declaration of Helsinki.

### 2.2. Anthropometric Data

Body mass and height were assessed in underwear and without shoes using a scale with an accuracy of 100 g and 0.5 cm, respectively. Waist circumference (WC) was measured midway between the lower edge of the last palpable rib and the top of the iliac crest using a nonstretchable tape measure. Body mass index (BMI) was calculated by dividing body weight (in kilograms) by the square of height (in meters), and waist-to-height ratio (WHtR) was calculated by dividing waist circumference (in centimeters) by height (in centimeters) [16]. An empirical mathematical model based on the association of anthropometric measurements (WC and BMI) and laboratory parameters (lipidogram) was used to assess the cardiometabolic risk [including ABSI, VAI, LAP, and BRI]. The VAI and LAP were calculated using sex-stratified formulas.

ABSI = WC/[BMI2/3 × Height1/2]

BRI = 364.2 − 365.5 × √(1 − [WC [cm]/2π]2/[0.5 × height cm]2)

DAI = {WC/[24.02 + (2.37 × BMI)] × [TG [mmol/L]/1.32] × [1.43/HDL-C [mmol/L]]}

LAP for women = (WC [cm] − 58) × (TG [mmol/L])

LAP for men = (WC [cm] − 65) × (TG [mmol/L])

VAI for women = [WC/36.58 + (1.89 × BMI)] × (TG [mmol/L]/0.81) × (1.52/HDL [mmol/L])

VAI for men = [WC/39.68 + (1.88 × BMI)] × (TG [mmol/L]/1.03) × (1.31/HDL [mmol/L])

### 2.3. Laboratory and Clinical Data

Biochemical data were obtained from patients’ medical records using the most recent laboratory test. Blood pressure (BP) measurements were performed according to the guidelines of the European Society of Hypertension using a digital electronic tensiometer (Omron Corp., Kyoto, Japan) [17].

MetS was defined according to the National Cholesterol Education Program Adult Treatment Panel III (NCEP ATP III) criteria. Participants were classified as having MetS if they had at least three of the following components: abdominal obesity (waist circumference ≥ 102 cm in men or ≥88 cm in women), triglycerides ≥ 150 mg/dL, HDL cholesterol < 40 mg/dL in men or <50 mg/dL in women, blood pressure ≥ 130/85 mmHg or taking antihypertensive medication, and fasting glucose ≥ 100 mg/dL or previously diagnosed diabetes [18].

### 2.4. Dietary Assessment

Dietary macronutrient intake was assessed using a seven-day food diary. Each participant received detailed instructions on accurately recording food and beverage consumption during the study period, including portion sizes and preparation methods. The collected dietary records were analyzed using commercial software Kcalmar.pro (Hermax, Lublin, Poland; 2025 version), which includes a Polish database of nutritional values for food products.

### 2.5. Statistical Analyses

For quantitative variables, the mean and standard deviation were calculated; for qualitative or ordinal variables, the percentage of each category was calculated. To determine whether there were differences in the analyzed variables across groups, the Mann–Whitney U test was used for quantitative variables, and the chi-squared test was used for qualitative or ordinal variables. Associations between quantitative variables were evaluated with Spearman’s rank correlation coefficient. A multivariable linear regression model was used to check the association between clinical and metabolic variables and the OARSI score. The analyses were performed using Statistica 13 PL (TIBICO Software Inc., Palo Alto, CA, USA) and PS IMAGO PRO 10 (IBM SPSS Statistics 29, IBM Corp., Armonk, NY, USA). A *p*-value of <0.05 was set as the level of statistical significance.

## 3. Results

Detailed characteristics of both groups are presented in Table 1. The mean age was 66.8 ± 7.5 years. The study group included 78 women (72.2%) and 30 men (27.8%). KOA patients had a statistically higher prevalence of obesity than the control group (*p* = 0.0051). Only 13.9% of KOA patients had a normal body weight, whereas over 85% were classified as overweight or obese. In contrast, obesity was less common in the control group (18.2%), but 60% of participants were overweight. The percentage of individuals with normal body weight was higher in the control group (21%) than in the KOA group. Among the anthropometric and metabolic indicators, BRI and LAP were significantly higher in KOA patients than in the control group (*p* = 0.0024 and *p* = 0.0290, respectively). No significant differences in ABSI values were found between the groups.

The mean OARSI score was 9.5 ± 3.2, and nearly 65% of patients had stage 3 Kellgren–Lawrence KOA. No significant differences were found between the groups in energy or macronutrient intake. In both groups, the proportions of energy from fat (34%) and saturated fat (11–12%) were relatively high compared with recommended dietary guidelines.

Approximately two-thirds of the KOA patients met the criteria for metabolic syndrome, and 70% were treated for hypertension. There was no statistically significant difference in the percentage of patients with and without MetS (*p* = 0.1135). Despite differences in anthropometric parameters, no significant differences were observed in biochemical parameters (glucose, lipids, and CRP) between the groups with and without MetS.

Spearman correlation analysis (Table 2) showed a moderate positive correlation between age and the OARSI score (r = 0.350, *p* < 0.001). In addition, weak but significant positive correlations were observed between the OARSI score and WHtR (r = 0.201, *p* = 0.037), serum glucose (r = 0.197, *p* = 0.041), and BRI (r = 0.201, *p* = 0.037). There was no significant association between BMI and OARSI score. Interestingly, both BMI and WHtR correlated positively with CRP (r = 0.257, *p* = 0.007; r = 0.244, *p* = 0.011, respectively), suggesting an association between obesity and low-grade inflammation. No significant correlations were found between disease duration and metabolic parameters, including glucose and lipid profile, in the overall group or in the gender subgroups (*p* > 0.05).

In multivariate linear regression, only age was associated with higher OARSI scores (β = 0.403, *p* < 0.001) (Table 3). The unstandardized coefficient indicated that each additional year of age was associated with an increase of approximately 0.17 points in OARSI scores. No significant correlations were observed for other parameters, such as WHtR (*p* = 0.051), C-reactive protein (*p* = 0.271), glucose (*p* = 0.788), total cholesterol (*p* = 0.739), or triglycerides (*p* = 0.463). The R2 for the model was 0.18, indicating that the variables included explained 18% of the variance in the dependent variable, the OARSI score.

After stratifying patients by disease duration (≤5 years vs. >5 years), a significantly higher prevalence of hypertension (80.8% vs. 58.9%, *p* = 0.0138) and MetS (73.1% vs. 55.4%, *p* = 0.055) was observed in patients with longer disease durations. These associations were particularly evident in women, in whom both hypertension (82.4% vs. 56.8%, *p* = 0.0166) and metabolic syndrome (82.4% vs. 54.5%, *p* = 0.0098) were significantly more common.

## 4. Discussion

This study assessed the relationships between metabolic factors, anthropometric, biochemical, and nutritional parameters in patients with KOA. Given the influence of metabolic disorders on KOA development, analyzing these factors and their interrelationships could help identify risk factors for cardiometabolic health and provide a basis for developing preventive strategies to protect joint cartilage.

In this study, the high prevalence of overweight and obesity was observed in the KOA group. These results are consistent with previous studies in the Polish population. For example, Stoś et al. analyzed the prevalence of abnormal body weight in a Polish adult population (n = 1831) and found that obesity was diagnosed in 16.4% of patients and overweight in 42.2% [19]. Similarly, Traczyk et al. reported that excess body weight affects almost 60% of Polish patients, increases with age, and poses a significant health challenge [20].

We also observed that the prevalence of obesity (*p* = 0.0051) and mean waist circumference (*p* = 0.0217) were higher in the study group than in the control group. This is consistent with other studies that emphasize that obesity is widely recognized as a major risk factor for both the development and progression of KOA. Obesity causes joint overload and impaired joint function, which contributes to reduced physical activity and, consequently, lower energy expenditure [21,22]. The risk of KOA increases significantly with BMI [23,24,25]. A meta-analysis describing the association between BMI and KOA showed that overweight individuals have an approximately 2.5-fold higher risk of developing KOA compared with individuals with a normal BMI [24]. Furthermore, Samma et al. found a statistically significant association between increased BMI and more severe radiographic features of KOA [26]. In our study, however, we found no significant association between BMI and the severity of structural changes in KOA patients (Table 2). However, WHtR showed a weak but statistically significant correlation with disease severity (ρ = 0.201, *p* = 0.037).

Although BMI remains the most popular tool for assessing obesity, it does not reflect body fat distribution as closely as waist circumference or WHtR. In our study, both parameters were higher in the KOA group than in the control group (Table 1). Furthermore, a weak positive correlation was observed between WHtR and the final OARSI score (r = 0.201, *p* = 0.037) (Table 2). These data are consistent with the notion that the metabolic effects of obesity are now considered as important as mechanical loading in contributing to OA development. The association between obesity and OA may be explained by the production of proinflammatory adipokines, which disrupt cartilage homeostasis and accelerate its degradation [27,28]. In the study by Lim et al., obesity, defined by high BMI and waist circumference, was associated with an increased risk of KOA progression [29]. Similarly, in a cross-sectional study by Phan et al., waist circumference, along with BMI, was a strong predictor of KOA severity in patients with KOA and metabolic syndrome. Furthermore, visceral obesity was also associated with a higher risk of cardiovascular disease and all-cause mortality [30].

In our study, we also analyzed parameters combining anthropometric and metabolic components, such as ABSI, BRI, LAP, DAI, and VAI. Analysis of these parameters revealed that KOA patients had significantly higher LAP scores than the control group (Table 1). Elevated LAP scores indicate excessive body fat accumulation and are a reliable predictor of chronic diseases, including metabolic syndrome, adverse cardiovascular events, and type 2 diabetes [31,32]. This is supported by NHANES data, which showed a positive correlation between LAP and OA, suggesting a strong association between visceral fat and joint health [33,34]. Furthermore, recent data indicate that this risk marker may be useful in clinical practice for cardiovascular risk screening in primary care [35].

Not only the LAP index but also the BRI index was higher in patients with KOA. A study conducted in a similar group of patients (postmenopausal women with obesity) showed that the BRI index effectively predicted the presence of metabolic syndrome [36]. Furthermore, the BRI index was identified as an independent predictor of osteoarthritis risk among patients with diabetes or prediabetes [37]. A large US cohort study (n = 32,995 adults) showed an increasing trend in the BRI index over almost 20 years and a U-shaped relationship between the BRI index and all-cause mortality, suggesting that the BRI index may serve as a noninvasive screening tool for estimating mortality risk [38].

Although no statistically significant difference in MetS prevalence was observed between the study and control groups, a high proportion of patients met the criteria for MetS. Furthermore, 70% of KOA patients were treated for hypertension. These results are consistent with previous studies showing an association between OA and a higher prevalence of metabolic diseases [39,40,41]. In our cohort, a longer duration of KOA was associated with a higher prevalence of hypertension and metabolic syndrome, particularly among women. These findings are consistent with previous reports indicating an increased cardiometabolic risk in postmenopausal women [42,43,44]. Hormonal changes associated with menopause, particularly estrogen deficiency, promote visceral adiposity, insulin resistance, and chronic low-grade inflammation, which may contribute to both metabolic disorders and the progression of OA [45,46].

CRP values were within the normal range, which is typical for OA [47]. However, both BMI and WHtR correlated positively with CRP values. This may suggest an association between central obesity and low-grade systemic inflammation, which contributes not only to structural joint degradation but also to pain severity and functional impairment by promoting the production of pro-inflammatory cytokines and adipokines [48,49,50].

The interrelationships between MetS and KOA reported in the literature are conflicting. In this study, we found weak but significant positive correlations between OARSI final score and WHtR, BRI, and serum glucose levels (Table 2). Yoshimura et al. found that BMI was the only significant predictor of KOA, whereas metabolic factors, including systolic blood pressure, HbA1c, fasting glucose, and lipid parameters, showed limited or no independent associations with OA after adjusting for BMI [7]. Xie et al. demonstrated that metabolic syndrome was associated with an increased incidence of osteophytes but not with joint space narrowing [8]. Similarly, Jansen et al. reported that, among the components of metabolic syndrome, abdominal obesity, assessed by waist circumference, had the strongest association with progression of KOA [51]. These results highlight the role of visceral obesity in OA and suggest that clinical data may vary across populations, suggesting a genetic component.

The majority of patients had moderate KOA (grade 3 in 64.81%) on the Kellgren–Lawrence scale (Table 1). These data are consistent with the severity of KOA observed in the European population, where, in addition to grade 3, mild KOA (grade 2) is also common [52,53,54].

In multivariate linear analysis (Table 3), we found that age was the only independent predictor of KOA severity (β = 0.403, *p* < 0.001), which is consistent with the results of other epidemiological and clinical studies, which have shown that age is the strongest non-modifiable risk factor for KOA, alongside female gender [55,56,57]. Furthermore, the development and progression of joint degeneration are strongly age-dependent and are associated with cellular senescence and impaired cartilage repair (a progressive imbalance between cartilage degradation and regeneration) [58,59].

Our study also revealed abnormal dietary patterns in the analyzed groups. Mean total fat intake in both groups exceeded 34% of total energy intake, while saturated fat intake was higher than 10% of total energy intake. Most dietary recommendations suggest that total fat intake should be 20–40% of daily energy intake, with saturated fat intake at or below 10% or as low as possible [60,61,62]. World Health Organization guidelines emphasize the need to limit total fat intake to ≤30% of total energy intake to prevent unhealthy weight gain [61]. Polish dietary guidelines also recommend a lower fat intake for individuals with lower physical activity levels [62]. The association between high-fat intake and an increased incidence of cardiovascular and metabolic diseases suggests that the type and quality of fat consumed, rather than total fat intake per se, appear to have a greater impact on metabolic health [63,64,65,66].

From a clinical perspective, our data underscore the importance of central obesity and metabolic health in the treatment of KOA. Targeted lifestyle interventions, including a balanced diet, reducing visceral adiposity, and improving metabolic health, may be beneficial for joint and cardiovascular health.

### Strengths and Limitations

Our study, despite providing an important assessment of OA and MetS from a general practitioner’s perspective, has some limitations. First, the cross-sectional, observational design limits the ability to establish causal relationships between KOA severity and the assessed factors. Longitudinal studies are needed to better understand temporal and causal associations. Second, the sample size was moderate, and the relatively small control group may limit the ability to capture the full heterogeneity of the KOA population and detect subtle associations, especially in multivariate analyses. Third, the study was conducted in a single geographic and ethnic population (Caucasian patients from a single region in Poland). Fourth, dietary intake was assessed using self-reported food diaries, which are prone to recall bias and underreporting. Furthermore, although numerous metabolic parameters were analyzed, the study did not include direct measurements of insulin resistance or inflammatory cytokines, which could provide further insight into the metabolic mechanisms underlying OA.

Nevertheless, our analysis of risk factors and metabolic health in patients with KOA in a primary care setting provides important information on the comorbidity of these two common diseases in the general population: osteoarthritis and obesity. This study, based on clinical and radiographic assessment of KOA, anthropometric and biochemical analyses, and dietary pattern assessment, provides comprehensive insight into the nature of KOA. The inclusion of traditional anthropometric measurements, visceral adiposity, and metabolic risk parameters (BMI, WHtR, waist circumference, ABSI, VAI, LA, DAI, and BRI) enabled a more accurate characterization of metabolic syndrome. These analyses were combined with a detailed assessment of structural OA severity (Kellgren–Lawrence OA scale and OARSI) and dietary intake (7-Day Dietary Assessment), providing valuable insights into the clinical and nutritional aspects of patients with KOA. This multifaceted approach allowed us to delineate the complex relationships between metabolic syndrome and KOA and identify parameters most strongly associated with metabolic risk, which can be used in clinical practice for patients with one of the most common degenerative diseases. Such analysis can help general practitioners and family doctors understand the complex nature of KOA and metabolic health, enabling them to treat both conditions effectively.

## 5. Conclusions

Our multifaceted study, based on clinical and radiographic assessment of KOA, anthropometric and biochemical analyses, and assessment of dietary habits, provides comprehensive insight into the nature of this disease. This study highlights not only the role of body weight but also the role of adipose tissue distribution in the progression of KOA. We demonstrated that the prevalence of central obesity was higher in KOA patients than in the control group. Furthermore, higher fasting glucose levels and WHtR were associated with greater radiographic severity of KOA, whereas age was the only independent predictor of structural KOA progression. Early detection of metabolic abnormalities in KOA using novel anthropometric indices enables the identification of patients at the highest cardiometabolic risk. Such assessment is possible in primary care and permits the planning of effective strategies for the prevention and treatment of metabolic disorders in KOA patients.

## Figures and Tables

**Table 1:** Characteristics of the study (patients with knee osteoarthritis) and the control group.

	KOA Patients (n = 108)	Control Group (n = 33)	*p* Value
Age (years)	6.8 ± 7.5	65.3 ± 7.6	0.1708
Gender (woman)	78 (72.2%)	22(6.7%)	
Current smoker	11 (10.2%)	3 (9.1%)	0.8407
Non-smoker	97 (89.8%)	30 (90.9%)	
SBP (mmHg)	137 ± 14.9	131.8 ± 10.9	0.0637
DBP (mmHg)	82.3 ± 9.3	81.6 ± 8.6	0.8662
Disease duration (years)	7.1 ± 8.1	-	-
BMI (kg/m^2^)	29.9 ± 4.7	28 ± 5.4	0.0061
Normal body mass (% of patients)	13.9%	21.2%	0.0197
Overweight	40.7%	60.6%	
Obesity	45.4%	18.2%	
WC (cm)	104.7 ± 1.8	9.5 ± 14.2	0.0217
WHtR	0.63 ± 0.07	0.59 ± 0.08	0.0025
ABSI	0.08 ± 0.00	0.08 ± 0.00	0.4065
BRI	6.21 ± 1.65	5.43 ± 2.03	0.0025
DAI	1.39 ± 0.94	1.15 ± 0.62	0.2485
LAP	77.12 ± 46.95	59.44 ± 34.93	0.0290
VAI	2.36 ± 1.52	1.93 ± 1.06	0.1870
Metabolic parameters			
Fasting blood glucose mg/dL	105.6 ± 2.4	103.4 ± 13.6	0.3050
Total Cholesterol (mg/dL)	201.5 ± 41.7	208.6 ± 43.1	0.3193
HDL (mg/dL)	57.8 ± 14.4	59.6 ± 12.3	0.5672
LDL (mg/dL)	116.0 ± 36.9	124.2 ± 39.5	0.2011
Triglycerides (mg/dL)	149.2 ± 72.2	135.8 ± 82.8	0.3123
CRP mg/L	2.3 ± 1.6	2.1 ± 1.8	0.4393
Metabolic syndrome *	69 (63.9%)	16 (48.5%)	0.1135
Hypertension	76 (70.4%)	19 (57.6%)	0.1701
Gout	6 (5.6%)	1 (3%)	0.5589
Diabetes	23 (21.3%)	6 (18.2%)	0.6985
OARSI score	9.5 ± 3.2	-	
Kellgren–Lawrence scale			
Grade 1	2 (1.85%)	-	
Grade 2	16 (14.81%)	-	
Grade 3	70 (64.81%)	-	
Grade 4	20 (18.52%)	-	
Patients after knee replacement Patients undergoing rehabilitation	14 (12.96%) 35 (32.4%)	- -	
Dietary habits			
Energy (kcal)	2190 ± 451	2168 ± 462	0.7204
Total protein intake (g/day) % of energy from protein	96.4 ± 2.6 17.7 ± 2.3	96.5 ± 19.1 18,9 ± 3.9	0.7131 0.7113
Total carbohydrate intake (g/day) % of energy from carbohydrates	248 ± 61.1 45.2 ± 6.1	245.4 ± 58 45.1 ± 3.9	0.6683 0.5821
Total fat intake (g/day) % of energy from fat	83.4 ± 21.4 34.3 ± 5.0	82.3 ± 20.5 34.1 ± 3.9	0.7059 0.8341
% of energy from saturated fat	1.5 ± 2.3	1.6 ± 2.3	0.9224
% of energy from simple sugars Total dietary fiber intake (g)	14.0 ± 4.2 25.7 ± 6.5	12.7 ± 3.7 26.6 ± 8.1	0.1682 0.5235

ABSI, body shape index; BMI, body mass index; BP, blood pressure; BRI, body roundness index; CRP, C-reactive protein; DAI, dysfunctional adiposity index; HDL, high-density lipoprotein; LAP, lipid accumulation product; LDL, low-density lipoprotein; KOA, knee osteoarthritis; VAI, visceral adiposity index; WHtR, waist-to-height ratio; WC, waist circumference; * according to the National Cholesterol Education Program Adult Treatment Panel III (NCEP ATP III) criteria. Data are expressed as mean ± standard deviation or n (%); *p*-values are from *t*-test, chi-squared test, or Mann–Whitney U test.

**Table 2:** Spearman correlations between clinical and metabolic variables and OARSI score in patients with knee osteoarthritis.

	Spearman’s Correlation Coefficient (ρ)	*p*
Age	0.350	<0.001
WHtR	0.201	0.037
Glucose	0.197	0.041
BMI	0.141	0.147
TGs (mg/dL)	0.107	0.269
CH (mg/dL)	−0.135	0.163
HDL (mg/dL)	0.032	0.740
Non-HDL (mg/dL)	−0.135	0.165
LDL (mg/dL)	−0.142	0.143
CRP (mg/L)	0.181	0.061
BRI	0.201	0.037
ABSI	0.096	0.324
VAI	0.099	0.307
LAP	0.162	0.094
DAI	0.084	0.388

BMI, body mass index; CH, total cholesterol; CRP, C-reactive protein; HDL, high-density lipoprotein; Non-HDL, non-high-density lipoprotein; LDL, low-density lipoprotein; TGs, triglycerides; WHtR, waist-to-height ratio.

**Table 3:** Multivariable linear regression model for OARSI score in patients with knee osteoarthritis.

	B	SE	β	t	*p*
Age (years)	0.173	0.042	0.403	4.097	<0.001
WHtR	8.538	4.328	0.181	1.973	0.051
Glucose (mg/dL)	0.004	0.014	0.027	0.270	0.788
CRP (mg/L)	0.206	0.186	0.105	1.106	0.271
CH (mg/dL)	−0.003	0.008	−0.033	−0.334	0.739
TGs (mg/dL)	0.003	0.004	0.074	0.737	0.463
OB (mm/h)	−0.019	0.030	−0.061	−0.622	0.536

CH, total cholesterol; CRP, C-reactive protein; WHtR, waist-to-height ratio; TGs, triglycerides.

## Data Availability

The raw data supporting the conclusions of this article will be made available by the authors upon request.

## Abbreviations

The following abbreviations are used in this manuscript:

ABSI	body shape index
BMI	body mass index
BP	blood pressure
BRI	body roundness index
CH	total cholesterol
CRP	C-reactive protein
DAI	dysfunctional obesity index
GPD	Global Burden of Disease, Injuries, and Risk Factors Study
Glc	glucose
HDL	high-density lipoproteins
KOA	knee osteoarthritis
LAP	lipid accumulation product
LDL	low-density lipoprotein
MetS	metabolic syndrome
NCEP ATP III	National Cholesterol Education Program Adult Treatment Panel III
OA	osteoarthritis
OARSI	Osteoarthritis Research Society International
TGs	triglycerides
VAI	visceral adiposity index
WC	waist circumference
WHtR	waist-to-height ratio
